# Investigating the mechanisms by which bisphenol A affects osteoarthritis through a novel network toxicology framework and experimental validation

**DOI:** 10.1186/s40360-026-01108-0

**Published:** 2026-02-25

**Authors:** Qiwang He, Shanlang Li, Yining Chen, Yue Liu, Yang Li, Shuqi Min, Shenlong Yi, Feng Li, Xiaohong Zhou

**Affiliations:** 1 Hubei University of Chinese Medicine, Wuhan, 430061 China; 2 Hubei Provincial Hospital of Traditional Chinese Medicine, Affiliated Hospital of Hubei University of Chinese Medicine, Hubei Key Laboratory of Theory and Application Research of Liver and Kidney in Traditional Chinese Medicine, Hubei Province Academy of Traditional Chinese Medicine, Wuhan, 430061 China; 3 Hubei Shizhen Laboratory, Wuhan, 430061 China; 4 Key Laboratory of Clinical Cohort Research on Bone and Joint Degenerative Diseases of Guangxi, Affiliated Hospital of Youjiang Medical University for Nationalities, Baise, 533000 China; 5 Fujian University of Traditional Chinese Medicine, Fuzhou, 350108 China; 6 General Hospital of Western Theater Command, Chengdu, 610083 China; 7 Changzhou Hospital of Traditional Chinese Medicine, Affiliated Hospital of Nanjing University of Chinese Medicine, Changzhou, 213000 China

**Keywords:** Bisphenol A, Osteoarthritis, Network toxicology, Molecular dynamics simulation, Experimental validation

## Abstract

**Background:**

In this study, we adopted network toxicology approaches to explore the potential risk effects of bisphenol A (BPA) on osteoarthritis (OA) processes.

**Methods:**

Targets related to BPA were obtained from the ChEMBL, Swiss Target Prediction, and STITCH databases, and OA-related targets were obtained from the GeneCards, DisGeNET, and OMIM databases; using the obtained information, we identified common targets. Then, the core targets were determined using the STRING database and the Cytoscape software, and functional enrichment analysis was subsequently conducted to elucidate the potential mechanisms. Moreover, a comprehensive validation of the core targets was conducted through molecular docking and dynamics simulations. Moreover, clinical samples were collected for experimental validation.

**Results:**

A total of 88 overlapping targets were identified, and six core targets (SRC, ESR1, EGFR, PTGS2, PPARG, and HSP90AA1) were further screened. The results of the enrichment analysis revealed that the main pathways through which BPA affects OA involve key signaling cascades, including the estrogen signaling pathway, the thyroid hormone signaling pathway, and the ErbB signaling pathway. The results of molecular docking and dynamic simulations indicated that there are stable binding interactions between BPA and the core targets. The results of the RT-qPCR and IHC assays revealed significant differences in the core targets between the OA group and the normal group.

**Conclusions:**

This study links the environmental toxin BPA with OA, systematically describing potential core targets and pathways. These findings emphasize the importance of reducing BPA exposure in public health, providing new insights for the formulation of subsequent environmental policies.

**Trial registration:**

Not applicable.

**Supplementary Information:**

The online version contains supplementary material available at 10.1186/s40360-026-01108-0.

## Background

The production and consumption of plastics have increased over recent decades. This is accompanied by the release of chemical substances during the use of plastic products, which poses hazards to the environment and human health [1]. Among these, bisphenol A (BPA), a key plastic chemical with a high production volume and extensive applications, is extensively used in the manufacture of detergents, plastic packaging, and food can coatings, among other products. This widespread use has exacerbated its extensive distribution in the environment. BPA is ubiquitous in water sources, drinking water, rivers, oceans, industrial wastewater, and even soil [2], subjecting humans to continuous exposure to this substance. Some studies have confirmed that BPA possesses endocrine-disrupting properties [3]. Therefore, fully understanding and assessing the potential threats of BPA to human health is highly important to prevent diseases and manage health.

BPA is an environmental contaminant with endocrine-disrupting properties. It can competitively bind to estrogen receptor alpha (ERα) and estrogen receptor beta (ERβ), antagonizing the protective effects of estrogen on chondrocytes and thereby disrupting the normal function of the endocrine system [3]. BPA may affect bone metabolism [4] and joint health [5] through multiple pathways, including exerting toxic effects on bone cells by reducing the activity of bone morphogenetic protein-2 and alkaline phosphatase. BPA is closely associated with immune function and inflammatory responses. Exposure to BPA can induce oxidative stress, leading to a greater production of free radicals, which may further damage articular cartilage and surrounding tissues [6]. Chemical-related gene set enrichment analysis by Li et al. suggested that BPA may be significantly associated with osteoarthritis (OA) [7]. BPA has been detected not only in the serum of OA patients but also in the synovial fluid of patients undergoing knee replacement surgery. It has a concentration-dependent antagonistic effect on the protective effect of estrogen on chondrocytes, subsequently reducing the activation of NF-κB and the expression of MMP1 [7]. These studies suggest that BPA may act as a contributing factor promoting the development of OA [8] and leading to further cartilage destruction and inflammatory responses [9]. Nevertheless, the molecular mechanisms that drive this process are still not well comprehended.

As the most common degenerative joint disease, OA causes functional impairment and disability, profoundly reducing the quality of life of affected individuals [10]. Its detrimental effects extend beyond individuals [11] to create significant healthcare burdens [12], as suggested by about 595 million global OA cases in 2020 [13]. Moreover, about 40% of OA patients are metabolically unhealthy, and this population has a significantly higher risk of mortality [14]. Since conventional treatments cannot alter the progression of OA, early prevention is crucial. The etiology of OA encompasses complex genetic, biological, biomechanical, and environmental determinants, among which environmental triggers have attracted the attention of researchers [15]. While studies indicate that BPA may contribute to OA, specific mechanistic insights are lacking. Therefore, elucidating the toxic mechanism of BPA on OA is of great significance for the early prevention, diagnosis, and subsequent treatment of this disease.

Network toxicology is an emerging branch of systems biology that extends beyond singular experimental observations [16]. It integrates chemicals, toxic phenotypes, and biological targets into a complex interaction network by leveraging multi-source heterogeneous databases [17]. Due to its efficiency, robustness, and broad applicability, it has become a key tool in environmental toxicology research. In this study, we established an integrated analytical framework combining network toxicology, molecular docking, and molecular dynamics (MD) simulations. This framework utilizes the multidimensional analytical capabilities of machine learning models to achieve correlative validation from macroscopic systemic levels down to microscopic physicochemical levels [18]. Through this integrated strategy, we systematically dissected the complex regulatory network through which BPA exposure influences OA, elucidated key toxic targets and mechanisms of action to a certain extent, provided relatively novel theoretical foundations for preventing and controlling health risks associated with environmental pollutants, and offered new insights into OA treatment.

## Materials and methods

**Fig. 1:**
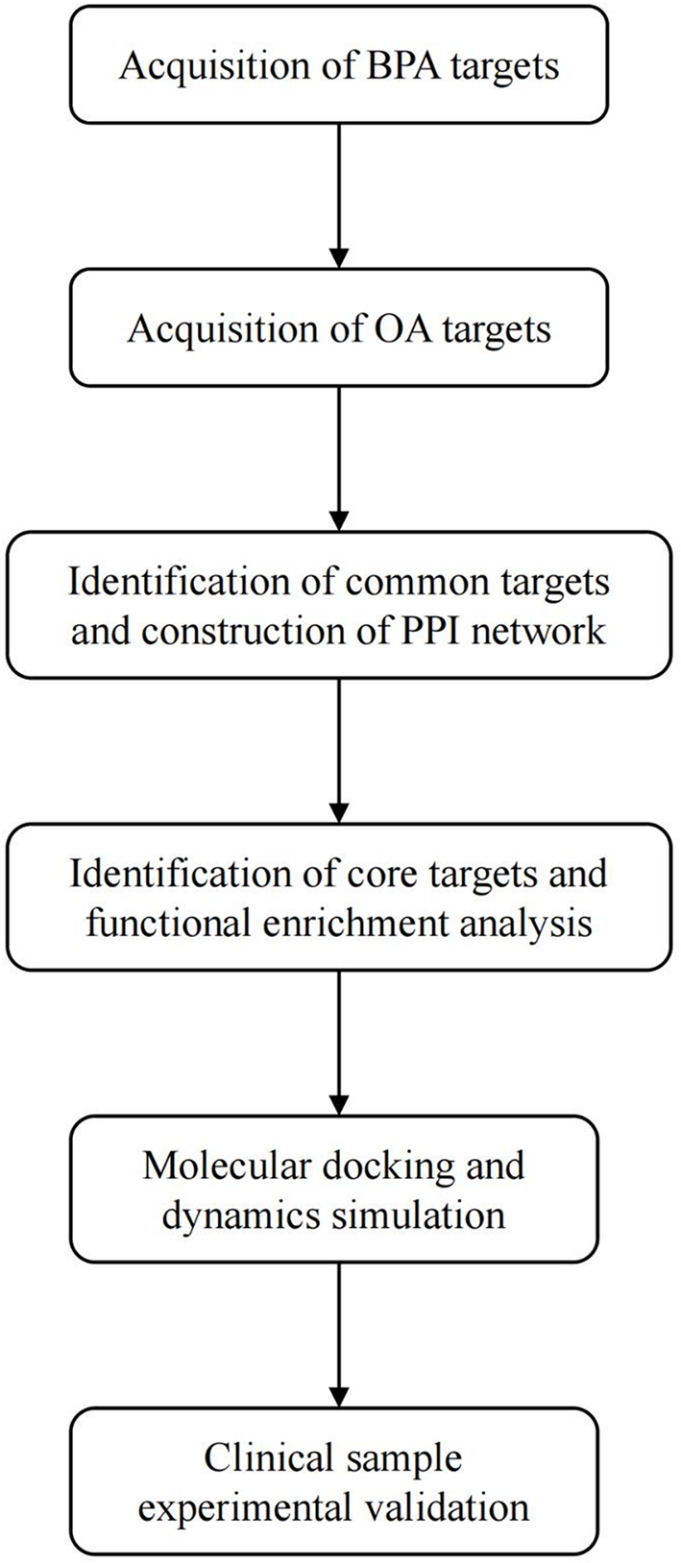
Overview of the research methodology

The research design is illustrated in Fig. 1.

### Collection of BPA targets

The standard structure and SMILES notation of BPA were queried through the PubChem database [19]. Based on the search results, a comprehensive BPA target library was integrated from the ChEMBL [20], Swiss Target Prediction [21], and the STITCH [22] databases, including all integrated and validated targets [23].

### Selection of the OA-related target network

We used the GeneCards [24], DisGeNET [25], and OMIM [26] databases to search for OA-related targets and integrated targets from various sources to remove duplicate data. Then, a specialized OA target library was established. Next, Venn diagram were used to determine common potential targets between BPA and OA. The genes that exhibited overlap were designated as potential targets associated with BPA-induced OA [27].

### Construction of the PPI network and core target selection

The potential targets of BPA-induced OA were input into the STRING database [28], the “minimum required interaction score” was set to “medium confidence > 0.4” for analysis, and the data generated by the STRING database were imported into Cytoscape for visualization. The cytoHubba plugin was used to identify the top 10 core targets via five common algorithms, including Maximal Clique Centrality (MCC), Maximum Neighborhood Component (MNC), Degree, Closeness, and Stress, and the common core targets were further filtered out.

### Core target enrichment analysis

To investigate the biological roles of the core targets in BPA-induced OA, the R software “clusterProfiler” package was utilized to perform Gene Ontology (GO) and Kyoto Encyclopedia of Genes and Genomes (KEGG) enrichment analyses of the core targets. A thorough GO analysis was performed, encompassing biological processes (BP), cellular components (CC), and molecular functions (MF); meanwhile, pathways related to BPA-induced OA were identified through KEGG analysis [29]. The findings were illustrated through bar and bubble plots employing the “ggplot2” package, and statistical significance was indicated by adj. *P* less than 0.05.

### Molecular docking verification

The core target protein’s structure was retrieved from the RCSB PDB database [30], while the structure of BPA was sourced from PubChem. Subsequently, PyMOL software was uesd to remove water molecules and the initial ligand during the protein preprocessing phase, with the results being stored in PDB file format. Then, docking was performed via the online molecular docking website CB-Dock2 [31]. Ultimately, PyMOL was uesd to analyze and illustrate the binding conformation of the core target protein in relation to BPA.

### Molecular dynamics simulation

The MD simulations in this study were conducted using the Gromacs 2022.3. Small-molecule components in the simulation system were subjected to the following preprocessing workflow: GAFF force field parameters were assigned using AmberTools22, and hydrogenation, along with restrained electrostatic potential electrostatic charge calculations, were performed utilizing Gaussian 16 W. The derived partial charge data were incorporated into the topological definition of the system. All MD simulations were conducted under isothermal (300 K) and isobaric (1 bar) conditions. The Amber99sb-ildn force field served as the core force field, with the solvent environment modeled using the Tip3P water model. Overall system charge neutrality was achieved by adding an appropriate number of Na^+^ counterions [32]. The simulation protocol was executed sequentially as follows: (1) Energy minimization was performed using the steepest descent algorithm. (2) NVT ensemble equilibration for 100 ps (100,000 steps, coupling time constant of 0.1 ps). (3) NPT ensemble equilibration for 100 ps (100,000 steps, coupling time constant of 0.1 ps). (4) Unrestrained conditions MD simulation for 100 ns (5,000,000 steps, 2 fs timestep). After conducting the simulations, trajectory analysis was performed using the built-in tools within Gromacs. The key metrics calculated include the root mean square deviation (RMSD) and root mean square fluctuation (RMSF) of amino acid residues, solvation effects measured by the solvent-accessible surface area (SASA), the radius of gyration of the protein (Rg), and comprehensive analysis incorporating binding free energies computed using the molecular mechanics generalized born surface area (MM/GBSA) method and free energy landscape (FEL) data.

### Clinical sample collection

A total of 28 patients undergoing knee arthroscopy were enrolled. The OA group comprised 15 individuals (mean age 60.4 ± 4.2 years, 60% female), whereas the control group included 13 patients with acute meniscal tears (*n* = 6) or anterior cruciate ligament rupture (*n* = 7) (mean age 31.9 ± 7.0 years, 46% female). Body mass index did not differ between groups (*P* > 0.05). All participants fulfilled standard indications for diagnostic/therapeutic knee arthroscopy. Inclusion criteria: OA group: (i) Fulfilment of the American College of Rheumatology clinical plus radiographic criteria for knee OA (knee pain and osteophytes on radiographs plus at least one of the following: age ≥ 50 years, morning stiffness < 30 min, or crepitus on active motion); (ii) Kellgren-Lawrence (K-L) grade 2–3 with clinically significant pain; and (iii) Arthroscopic confirmation of marked synovial hypertrophy, with sampling of suprapatellar synovium. Control group: (i) Acute knee trauma (< 3 months); (ii) K-L grade 0–1; and (iii) Arthroscopic absence of macroscopic synovial hypertrophy or hyperaemia, with procurement of morphologically uninvolved suprapatellar synovium. Exclusion criteria: (i) Co-existing inflammatory arthropathies (rheumatoid arthritis, gout, infectious arthritis, pigmented villonodular synovitis, etc.); (ii) Previous ipsilateral knee surgery (meniscectomy, ligament reconstruction, etc.); and (iii) Severe systemic disease (malignancy, autoimmune disorders, etc.).

Five OA synovium samples and five normal synovium samples were randomly selected for RT-qPCR experiments; 10 OA synovium samples and eight normal synovium samples were used for immunohistochemistry (IHC) experiments. This study was approved by the Ethics Committee of the Affiliated Hospital of Youjiang Medical University for Nationalities, with informed consent obtained from all participants (Ethics No. 2025011301).

### RT-qPCR analysis

Five samples of synovial tissue from patients with knee OA and five samples of normal synovial tissue were collected. Total RNA was isolated from the samples utilizing TRIzol reagent (Thermo Fisher Sci, Inc.) and reverse-transcribed into cDNA. The thermal cycling conditions used were as follows: denaturation at 95 °C for 30 s, annealing at 95 °C for 3 s, and extension at 60 °C for 30 s. GAPDH was used as an endogenous control, and the results were calculated utilizing the 2^–ΔΔCt^ method. Table 1 provides an overview of the primers utilized in the current investigation.

**Table 1:** Primer sequences for RT-qPCR

Gene	Primer sequence (5’–3’)	Size (bp)
H-SRC-S	CTACTGCCTCTCAGTGTCTGACTTC	157
H-SRC-A	ATCGGCGTGTTTGGAGTAGTAG	
H-ESR1-S	CACTCAACAGCGTGTCTCCGA	239
H-ESR1-A	AGATTCCATAGCCATACTTCCCTT	
H-EGFR-S	TTGCCGCAAAGTGTGTAACG	112
H-EGFR-A	GAGATCGCCACTGATGGAGG	
H-PTGS2-S	GGGTTGCTGGTGGTAGGAATG	116
H-PTGS2-A	CATAAAGCGTTTGCGGTACTCAT	
H-PPARG-S	AGACCACTCCCACTCCTTTGAT	176
H-PPARG-A	GAGATGCAGGCTCCACTTTGAT	
H-HSP90AA1-S	GCCCAAGTGTTTCTCTGGCA	210
H-HSP90AA1-A	TTGGTCTTGGGTCTGGGTTTC	
H-GAPDH-S	GGAAGCTTGTCATCAATGGAAATC	168
H-GAPDH-A	TGATGACCCTTTTGGCTCCC	

### IHC analysis

In total, 10 OA and eight normal synovial samples obtained intraoperatively were immediately fixed in 10% neutral-buffered formalin and embedded in paraffin. For IHC analysis, 2–3 μm sections were cut, baked at 68 °C for 2 h, deparaffinized in xylene (20 min), and rehydrated through a graded ethanol series. After three rinses, antigen retrieval was performed in EDTA buffer utilizing a pressure cooker, followed by cooling to 30 °C. Endogenous peroxidase was blocked with 3% H_2_O_2_ for 10 min in a humidified chamber. The sections were incubated overnight at 4 °C with the following primary antibodies (all from Proteintech, diluted in PBS): SRC (1:200, 60315-1-Ig), ESR1 (1:200, 21244-1-AP), EGFR (1:1000, 18986-1-AP), PTGS2 (1:1000, 66351-1-Ig), PPARG (1:400, 16643-1-AP), and HSP90AA1 (1:400000, 13171-1-AP). After the slides were washed with PBS, they were treated with the appropriate HRP-conjugated secondary antibodies, developed with 3,3′-diaminobenzidine, and counterstained with hematoxylin. Finally, the sections were dehydrated through graded ethanol and xylene, mounted with neutral resin, and photographed under a light microscope. Staining was quantified with Image-Pro Plus 6.0 (Media Cybernetics, USA). The immunoreactive score (IRS) was calculated as the staining intensity × positive area percentage. The intensity was graded as negative (0), weak (1), moderate (2), or strong (3). The percentage of positive cells was scored as 0 (0%), 1 (1–25%), 2 (26–50%), 3 (51–75%), or 4 (76–100%). Total IRS of 0, 1–4, 5–8, and 9–12 were defined as negative, mildly positive, moderately positive, and strongly positive, respectively.

### Statistical analysis

Statistical analyses were carried out utilizing R software, and an independent samples t-test was employed to assess the variations in gene expression between the two groups. All differences were considered to be statistically significant at *P* less then 0.05.

## Results

### Identification of targets for BPA-induced OA

**Fig. 2:**
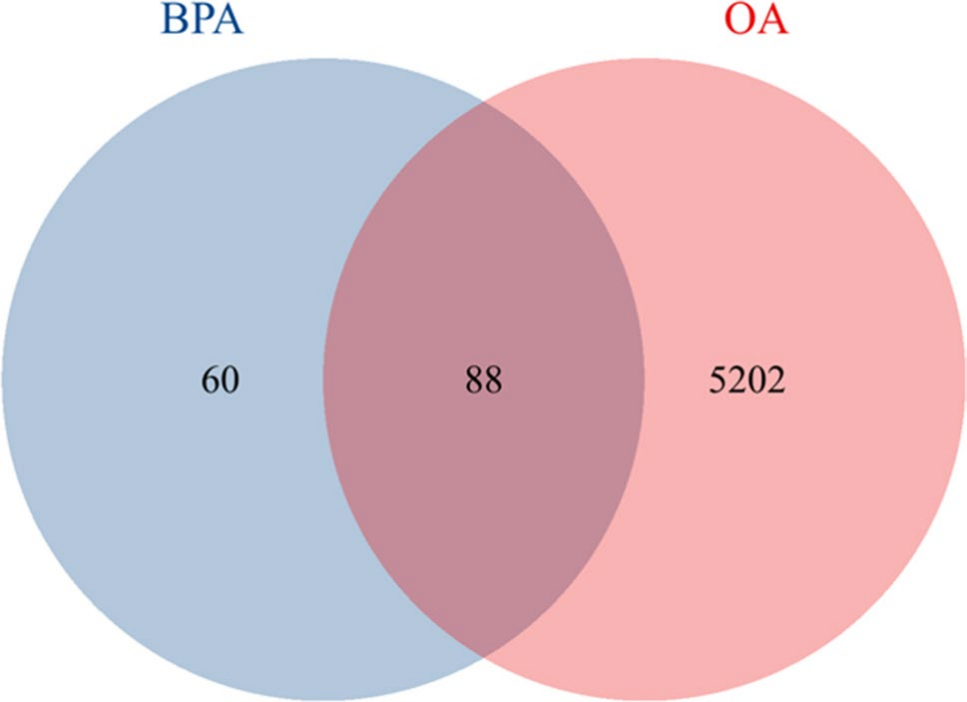
Venn diagram of the targets of BPA and OA

Using the ChEMBL, Swiss Target Prediction, and STITCH databases, 148 targets associated with BPA were identified. Additionally, an analysis of the GeneCards, DisGeNET, and OMIM databases revealed 5,290 targets associated with OA. A total of 88 common cross-targets shared by BPA and OA were subsequently identified (Fig. 2), and these intersecting targets are potential candidates for the influence of BPA on OA.

### Interaction network of common potential targets and acquisition of core targets

**Fig. 3:**
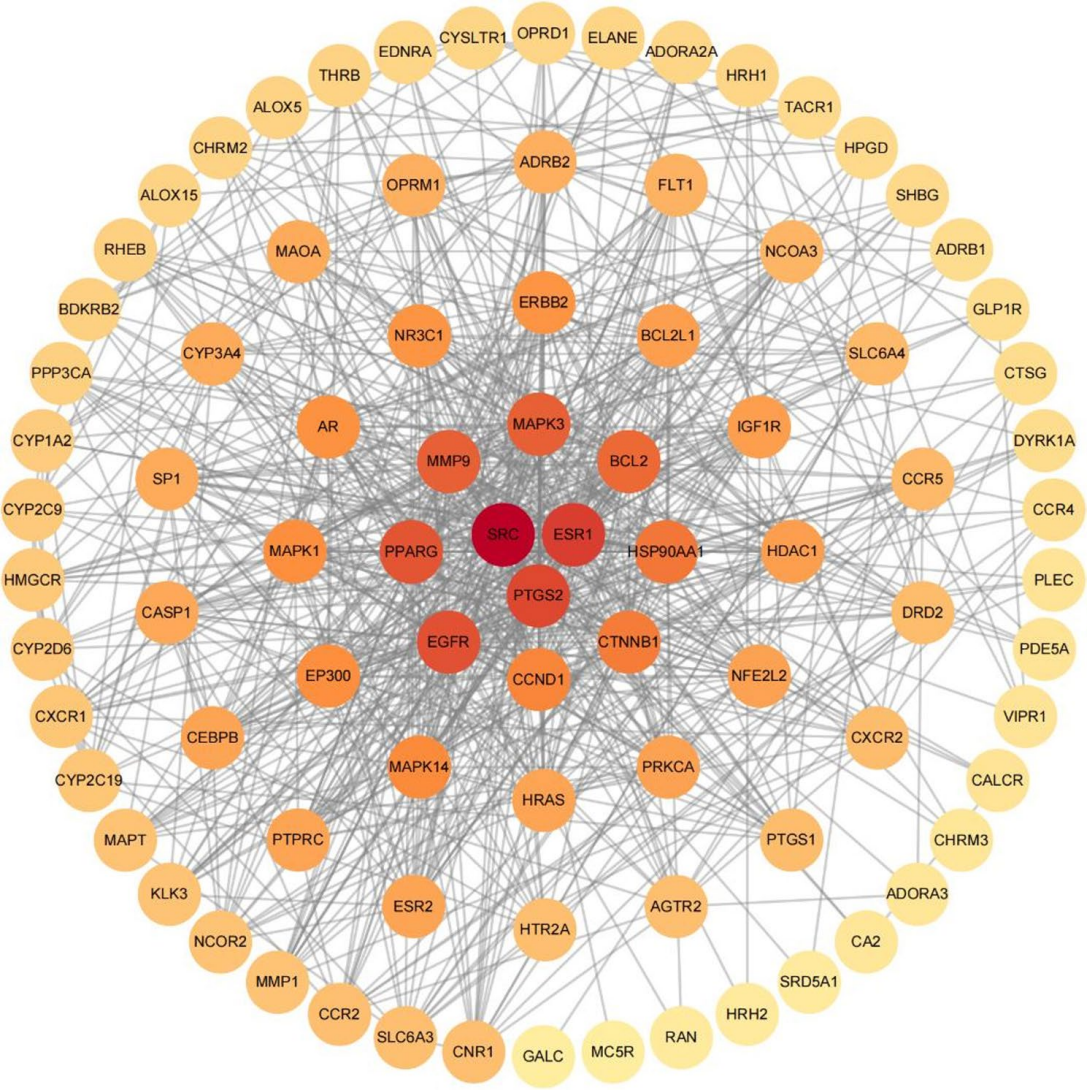
The PPI network of common potential targets

A PPI network with 87 nodes and 747 edges was generated using the STRING database (Fig. 3). The top 10 potential targets ranked by MCC (Supplementary Fig. 1A), MNC (Supplementary Fig. 1B), Degree (Supplementary Fig. 1C), Closeness (Supplementary Fig. 1D), and Stress (Supplementary Fig. 1E) were generated using the cytoHubba plugin. Finally, using five algorithms, six intersecting targets were obtained: SRC, ESR1, EGFR, PTGS2, PPARG, and HSP90AA1 (Supplementary Fig. 1F).

### GO and KEGG enrichment analyses of core targets

**Fig. 4:**
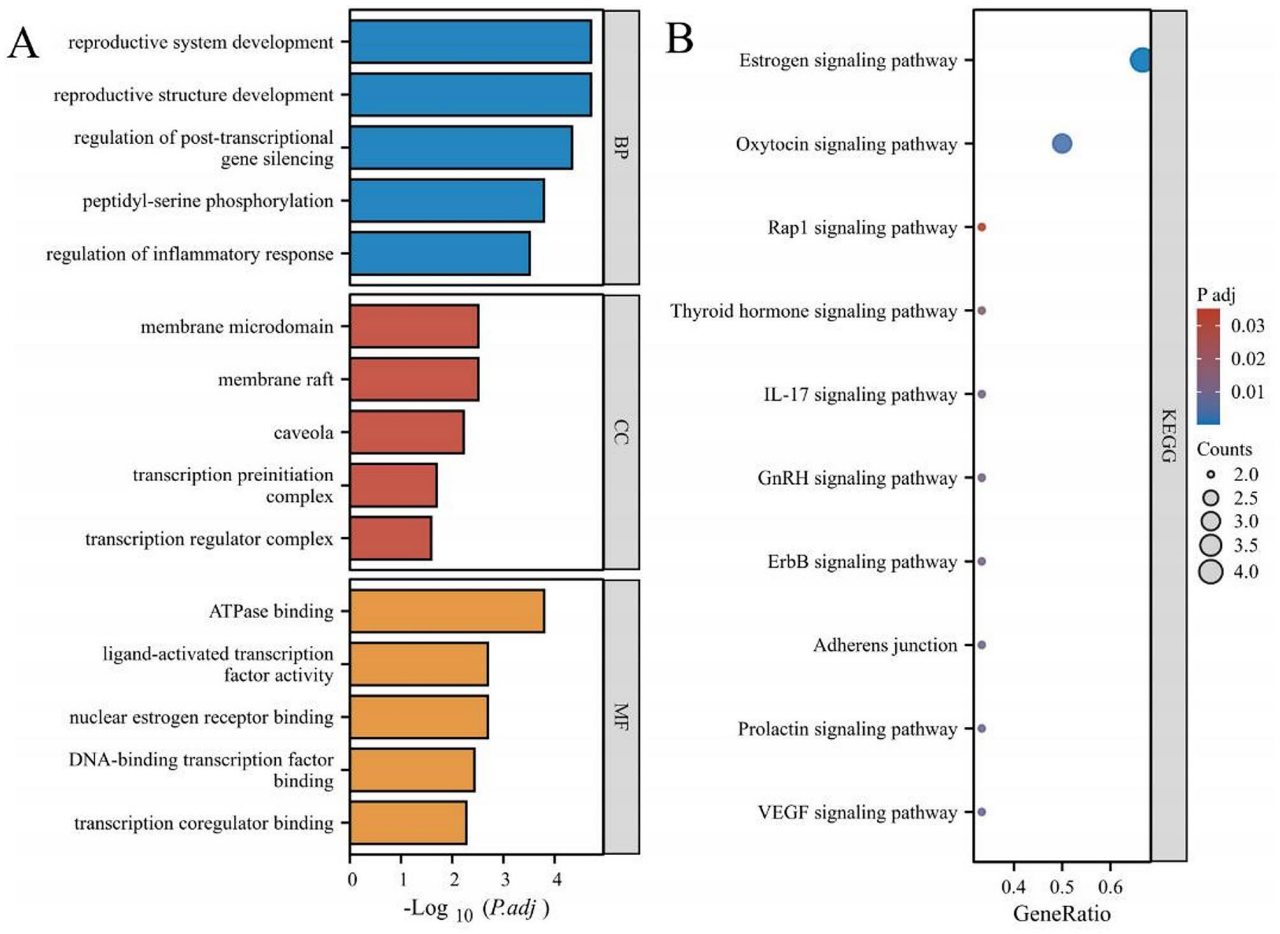
GO and KEGG enrichment analyses. (**A**) Bar diagram illustrating the results of the GO analysis. (**B**) Bubble diagram illustrating the results of the KEGG analysis

Enrichment analysis was performed on six core targets, resulting in 788 statistically significant GO entries, including 686 BP entries, 28 CC entries, and 74 MF entries. As shown in Fig. 4A, in the BP category, the targets were enriched mainly in reproductive structure development, the regulation of post-transcriptional gene silencing, and the regulation of inflammatory response; in the CC category, the targets were enriched mainly in the membrane raft, the membrane microdomain, and the transcription preinitiation complex; in the MF category, the targets were enriched mainly in the ATPase binding, the nuclear estrogen receptor binding, and the ligand-activated transcription factor activity.

Moreover, a total of 30 signaling pathways with statistically significant differences were enriched. The core targets of BPA and OA are related mainly to the estrogen signaling pathway, the thyroid hormone signaling pathway, and the ErbB signaling pathway (Fig. 4B).

### Molecular docking verification

**Fig. 5:**
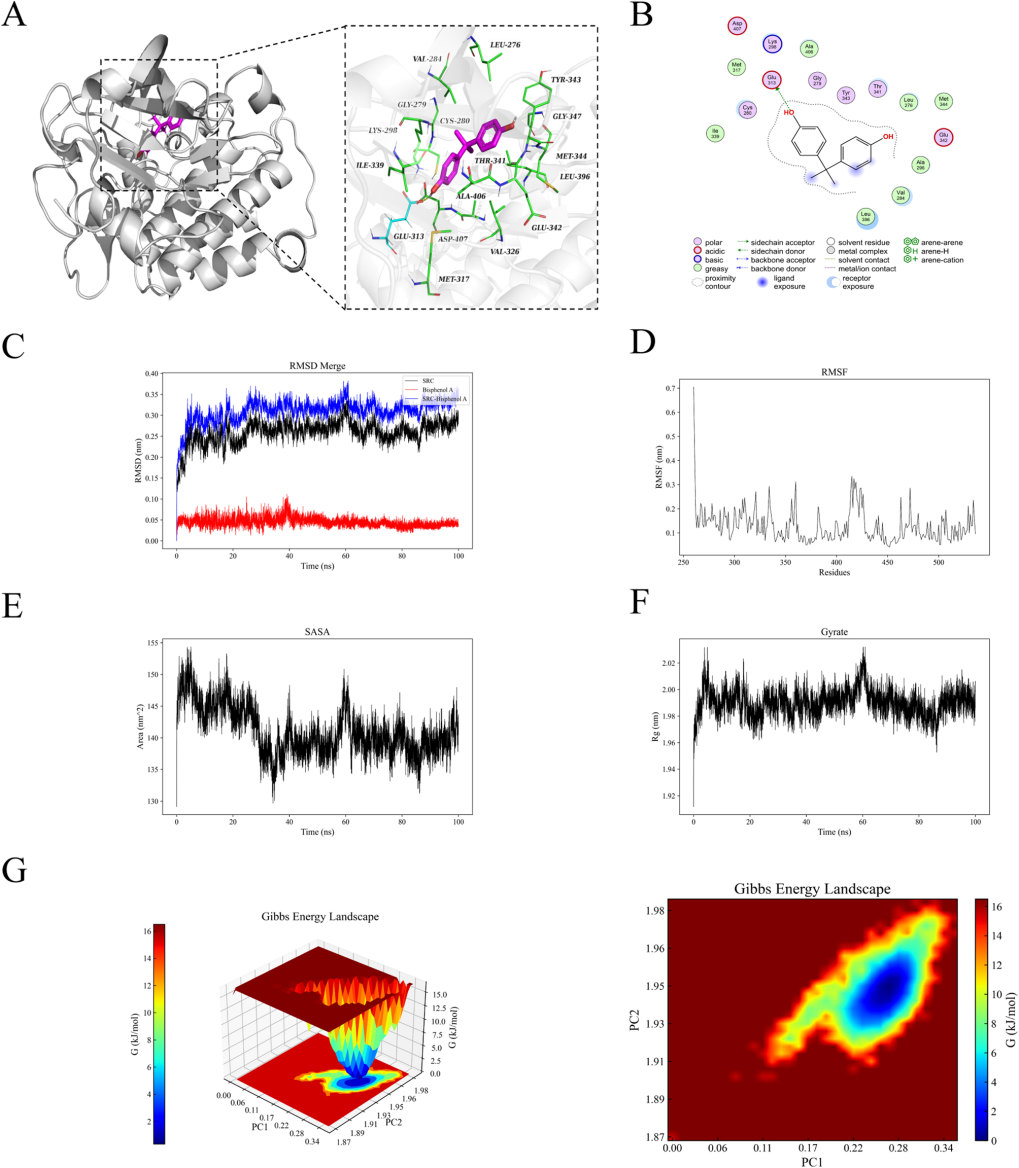
Molecular docking and dynamics simulation of BPA with SRC: (**A**) SRC-BPA-3D, (**B**) SRC-BPA-2D, (**C**) RMSD values of the SRC-BPA complex over time, (**D**) RMSF values of backbone atoms in the SRC-BPA complex over time, (**E**) SASA values of the SRC-BPA complex over time, (**F**) Rg values of the SRC-BPA complex over time and (**G**) FEL of the SRC-BPA complex

Molecular docking simulations were conducted to investigate the interactions between BPA and the six core target proteins. The results revealed binding energies ranging from − 5.9 to − 8.3 kcal/mol for the target proteins: SRC (–7.7 kcal/mol; Figs. 5A and B), ESR1 (–8.3 kcal/mol; Supplementary Figs. 2 A and B), EGFR (–6.9 kcal/mol; Supplementary Figs. 3 A and B), PTGS2 (–7.6 kcal/mol; Supplementary Figs. 4 A and B), PPARG (–7.9 kcal/mol; Supplementary Figs. 5 A and B), and HSP90AA1 (–5.9 kcal/mol; Supplementary Figs. 6 A and B). Notably, all six core targets exhibited strong binding affinities with BPA, with binding energies < − 5.0 kcal/mol [33]. According to established criteria [34], binding affinities < − 4.25 kcal/mol indicate standard binding capability, values < − 5.0 kcal/mol signify good binding affinity, and values < − 7.0 kcal/mol suggest strong binding activity. These energetically favorable interactions indicate stable binding between BPA and each core target, further supporting their significant mechanistic roles in BPA-induced pathogenesis of OA.

### Molecular dynamics simulation

To evaluate the binding stability and affinity of BPA with six target proteins, 100 ns MD simulations were performed on each complex. Key analyses included system stability metrics (RMSD: 0.2–0.35 nm (Fig. 5C, Supplementary Figs. 2–6 C); RMSF: 0.12–0.15 nm (Fig. 5D, Supplementary Figs. 2-6D); the SASA value decreased over the course of the simulation (Fig. 5E, Supplementary Figs. 2-6E); Rg: 1.7–2.4 nm (Fig. 5F, Supplementary Figs. 2–6 F); and binding free energy decomposition. All complexes maintained low structural fluctuations and compact folding throughout the simulations. Energy decomposition revealed favorable binding affinities (ΔTotal = − 29.37 to − 33.73 kcal/mol, Supplementary Table 1), with the PPARG-BPA complex exhibiting the strongest interaction (–33.73 ± 0.16 kcal/mol). The FEL describes the free energy profile of a protein-ligand complex under constant temperature and pressure, reflecting the stability of the system. In this study, the free energy landscapes of the docked complexes in all six systems consistently exhibited a single, sharp energy minimum, indicating that these complexes maintained a highly stable state throughout the simulation (Fig. 5G, Supplementary Figs. 2-6G). These findings suggest that the structural and functional integrity of these complexes can be preserved under physiological conditions, thereby increasing their biological activity. These findings indicate that BPA has favorable binding stability and affinity toward these target proteins, further validating the results of the molecular docking predictions. These findings provide a theoretical foundation for subsequent targeted intervention strategies and rational drug design.

### RT-qPCR analysis

**Fig. 6:**
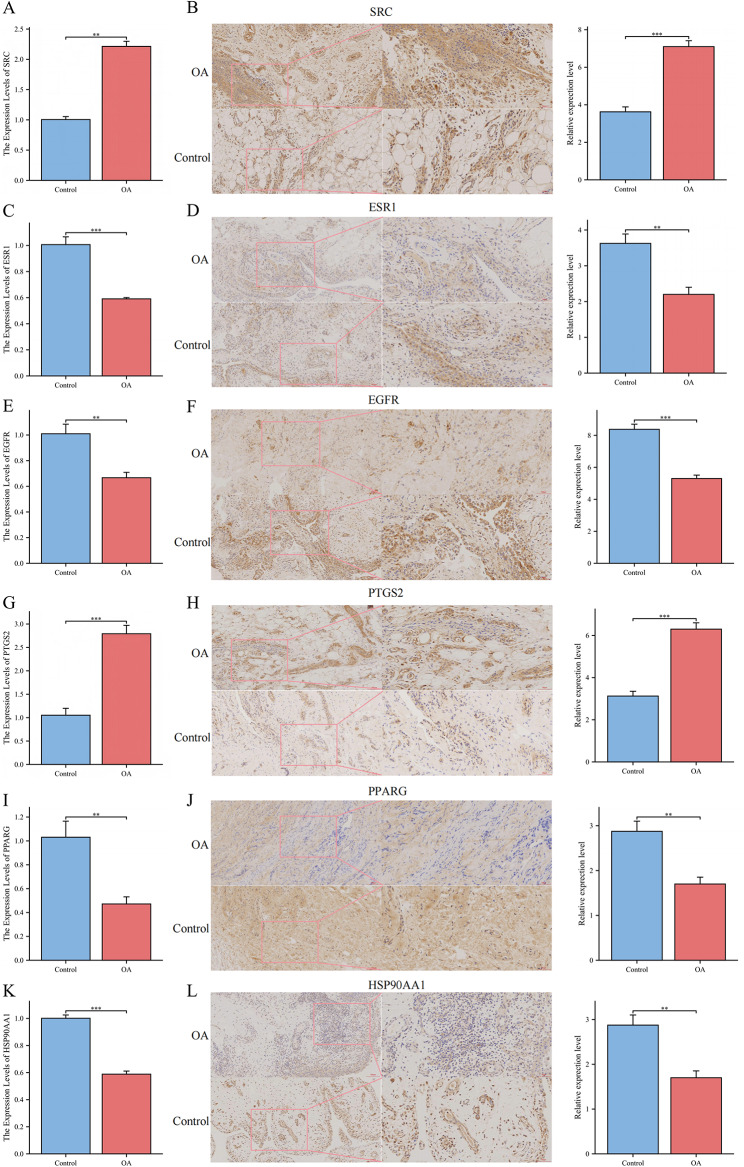
Experimental validation. (**A**,** C**,** E**,** G**,** I** and **K**) Relative expression of SRC, ESR1, EGFR, PTGS2, PPARG and HSP90AA1 via RT-qPCR. (**B**,** D**,** F**,** H**,** J** and **L**) Typical pictures and scores of the relative expression of SRC, ESR1, EGFR, PTGS2, PPARG and HSP90AA1 via IHC; 20×, Scale bar: 100 μm; 40×, Scale bar: 50 μm. ** *P* < 0.01, and *** *P* < 0.001

Using clinical tissue samples, we conducted RT-qPCR experiments to validate the expression of six genes. The findings indicated that compared to those in the normal group, the relative expression levels of SRC and PTGS2 were considerably higher in disease samples (Figs. 6A and G), whereas the relative expression levels of ESR1, EGFR, PPARG, and HSP90AA1 were significantly lower (Figs. 6C, E, I, and K).

### IHC analysis

When viewed under significant magnification, SRC and PTGS2 were strongly positive in the OA samples but weakly positive in the normal samples. ESR1, EGFR, PPARG, and HSP90AA1 were weakly positive in the OA samples but strongly positive in the normal samples. Compared to those in the normal samples, the relative expression levels of SRC and PTGS2 were significantly higher (Figs. 6B and H), whereas the relative expression levels of ESR1, EGFR, PPARG, and HSP90AA1 were significantly lower (Figs. 6D, F, J, and L).

## Discussion

We used network toxicology methods, integrating data from multiple databases and systematically identifying 88 common potential targets associated with BPA exposure and OA. A PPI network was constructed using the STRING database and Cytoscape software, which identified six core targets that play key roles in BPA-induced OA, including SRC, ESR1, EGFR, PTGS2, PPARG, and HSP90AA1, providing significant perspectives that contribute to a better understanding of their possible mechanisms.

SRC (Steroid receptor coactivator) is a non-receptor tyrosine kinase that is involved in regulating cell cycle progression. In OA, SRC plays a key role in the proliferation and differentiation of chondrocytes. The expression of SRC is significantly increased in the cartilage tissues of OA patients, whereas the phosphorylation of SRC is elevated in the synovial tissues of OA patients [35]. The use of SRC inhibitors such as PP2 can effectively reduce the degradation of the chondrocyte matrix and prevent further destruction of cartilage [36]. SRC signaling may also lead to further development of OA by promoting the production of matrix-degrading proteases, which in turn leads to the continued degradation of the cartilage matrix [37].

ESR1 (Estrogen receptor-1), which encodes ERα, is significantly downregulated in severely damaged cartilage. ESR1 may indirectly affect bone health by influencing estrogen-related metabolism and hormone levels [38]. The ESR1 gene is closely associated with various bone metabolism-related diseases, including OA. Modulation of ESR1 and its downstream signaling pathways can effectively mitigate inflammation and cartilage degradation induced by OA [39]. Chondrocytes depleted of ERα respond to detrimental mechanical loading by considerably increasing the expression of molecules associated with hypertrophy and osteogenesis [40]. Thus, by interacting with other key genes, ESR1 regulates the response of chondrocytes to mechanical stress and ultimately influences the progression of OA.

EGFR (Epidermal growth factor receptor), a 170-kDa kinase receptor, can regulate downstream signaling pathways after being activated by ligands, thereby regulating various cellular functions, including proliferation, migration, and differentiation [41]. EGFR has also been studied more extensively in OA. The activation of EGFR signaling is essential for stimulating the expression of proteoglycan 4, enhancing cartilage surface lubrication, and maintaining the integrity of the superficial zone, thereby protecting against OA. EGFR deficiency substantially reduces the cartilage surface modulus [42]. Some studies indicate that EGFR may serve as a therapeutic target for OA by enhancing lubricating function in articular cartilage [43]. The EGFR signaling pathway in chondrogenesis sustains postnatal slow-cycling cells and is greatly involved in maintaining adult cartilage homeostasis and driving the progression of OA.

PTGS2 (Prostaglandin endoperoxide synthase 2), also known as Cyclooxygenase-2, is involved in the synthesis of prostaglandin E2 (PGE2), which in turn is involved in the regulation of inflammation and pain. In the synovial tissues of OA patients, the expression of PTGS2 is significantly upregulated [44]. PGE2 plays a key role in the pathological processes of OA and rheumatoid arthritis (RA). By binding to EP receptors, it triggers downstream signal transduction (involving the cAMP/PKA and PLC/PKC pathways), thereby promoting the substantial release of inflammatory cytokines such as interleukin-6 (IL-6) and tumor necrosis factor alpha (TNF-α). This amplification of inflammatory signals directly leads to intensified synovial inflammation and accelerated degradation of the cartilage matrix, thereby exacerbating joint damage [45]. Additionally, PTGS2 responds to mechanical stress and is involved in inducing inflammatory responses to stimuli [46]. PTGS2 may be a Piezo1-related downstream gene in chondrocytes and may be involved in the formation and maturation of OA osteoids [47]. Interventions targeting PTGS2 and its downstream signaling pathway may provide more effective treatment options for patients with OA.

PPARG (Peroxisome proliferator-activated receptor gamma), an important transcription factor, plays an important role in lipid metabolism and the inflammatory response. In synoviocytes from OA patients, PPARG expression is downregulated, which may be closely related to abnormal lipid metabolism and inflammatory processes [48]. Additionally, PPARG inhibits the inflammatory response by regulating the expression of inflammatory factors (IL-6 and TNF-α), and PPARG deficiency promotes the expression of OA-related genes and exacerbates the inflammatory response [49]. PPARG activation has a protective effect on joints. In a mouse model of OA induced by destabilization of the medial meniscus, treatment with PPARG agonists ameliorated cartilage damage and promoted matrix synthesis [50].

HSP90AA1 (Heat shock protein 90AA1) is an important molecular chaperone protein that plays a key role in various cellular processes, including cell cycle control, cell survival, and signaling. Studies have shown that HSP90AA1 is downregulated in human OA cartilage and that there is a close association between HSP90AA1 expression and the risk of OA; moreover, HSP90AA1 deficiency leads to autophagy dysfunction, exacerbation of the inflammatory response, and an increase in oxidative stress, which then exacerbates the progression of OA [51]. Additionally, through microarray expression profiling of OA-related genes, HSP90AA1 was identified as an OA autophagy-related marker [52], and in-depth studies targeting the function and regulatory mechanism of HSP90AA1 may be helpful in the diagnosis of OA, as well as in designing immunotherapeutic strategies and individualized drug treatments.

Estrogen plays a key role in bone metabolism by regulating chondrocyte activity [53]. It enhances the synthesis of collagen II and aggrecan, which are essential for cartilage resilience. It simultaneously suppresses catabolic enzymes such as MMPs and ADAMTS to mitigate matrix degradation [54]. These effects of estrogen are mediated through estrogen receptors. Studies indicate that ERα, but not ERβ, is primarily responsible for the protective actions of estrogen in cartilage [54]. The net effect of estrogen depends on its concentration, receptor subtype dominance, and the cellular microenvironment [55]. BPA may disrupt this balance by acting as an ER antagonist. In contrast, the oxytocin signaling pathway serves as an endogenous protective mechanism in articular cartilage. The oxytocin receptor (OTR) is expressed in chondrocytes, and its expression is significantly reduced in OA [56]. The activation of OTR promotes anabolic activity by upregulating aggrecan and other matrix components through the MAPK and PKC signaling pathways [56]. Vascular endothelial growth factor (VEGF) signaling actively contributes to the progression of OA. VEGF levels are elevated in the synovium, cartilage, and synovial fluid of OA patients [57]. By activating VEGFR-2, VEGF induces angiogenesis and disrupts the avascular environment of cartilage, facilitating the infiltration of inflammatory cells and cytokines that exacerbate the destruction of joints [58].

Although there is no direct evidence definitively establishing a causal relationship between ATBC and OA, we propose the hypothesis that ATBC may exert potential adverse effects on OA, based on rigorous screening and prediction through network toxicology approaches, complemented by an analysis of real-world exposure situations. We emphasize the importance of considering all factors that might negatively influence the mechanisms of OA onset and progression associated with ATBC, in order to prevent unforeseen consequences.

### Limitations

This study still has certain limitations. First, the research is mainly based on network toxicology, molecular docking, and MD simulations, combined with a small-scale clinical sample for validation. There is a lack of direct experimental exploration and large-scale prospective cohort validation, and it is currently impossible to establish a direct causal relationship between BPA exposure and the occurrence and development of OA; the research results provide more theoretical basis and hypothetical direction for the potential association between the two. Second, the core targets selected in the study were obtained through topological centrality ranking, which may lead to a preferential selection of highly connected nodes, thus introducing a certain degree of algorithmic bias; in addition, molecular docking/MD/MM-GBSA calculations can only reflect the physical level of binding possibilities and cannot fully equate to the functional regulatory effects in vivo, and the specific functional impacts await further exploration in subsequent experiments.

## Conclusion

In conclusion, this study systematically explored the potential mechanisms by which BPA affects OA through network toxicology methods, identifying 88 overlapping targets and highlighting six core targets: SRC, ESR1, EGFR, PTGS2, PPARG, and HSP90AA1. These findings deepen the understanding of the pathogenesis mechanism of BPA exposure, emphasizing the urgent need to reassess the health risks associated with BPA exposure and providing new insights for the development of management strategies for OA prevention and treatment. However, further clinical research and experimental exploration are needed to validate these results.

## Supplementary Information

Below is the link to the electronic supplementary material.

Supplementary Material 1

## Data Availability

The datasets generated during and/or analysed during the current study are available from the corresponding author on reasonable request.

## Abbreviations

BPA: Bisphenol A
ERα: Estrogen receptor alpha
ERβ: Estrogen receptor beta
OA: Osteoarthritis
MD: Molecular dynamics
MCC: Maximal Clique Centrality
MNC: Maximum Neighborhood Component
GO: Gene Ontology
KEGG: Kyoto Encyclopedia of Genes and Genomes
BP: Biological process
CC: Cellular component
MF: Molecular function
RMSD: Root mean square deviation
RMSF: Root mean square fluctuation
SASA: Solvent accessible surface area
Rg: Radius of gyration
MM/GBSA: Molecular mechanics generalized born surface area
FEL: Free energy landscape
K-L: Kellgren-Lawrence
IHC: Immunohistochemistry
IRS: Immunoreactive score
PGE2: Prostaglandin E2
RA: Rheumatoid arthritis
IL-6: Interleukin-6
TNF-α: Tumor necrosis factor alpha
OTR: Oxytocin receptor
VEGF: Vascular endothelial growth factor
