# Supplementary material for: Investigating the mechanisms by which bisphenol A affects osteoarthritis through a novel network toxicology framework and experimental validation
Source: BMC Pharmacol Toxicol. 2026 Feb 25;27:50. doi: 10.1186/s40360-026-01108-0 (PMC13040831; doi:10.1186/s40360-026-01108-0)
Supplement: Supplementary file 1 — Supplementary Material 1 [file 40360_2026_1108_MOESM1_ESM.docx]

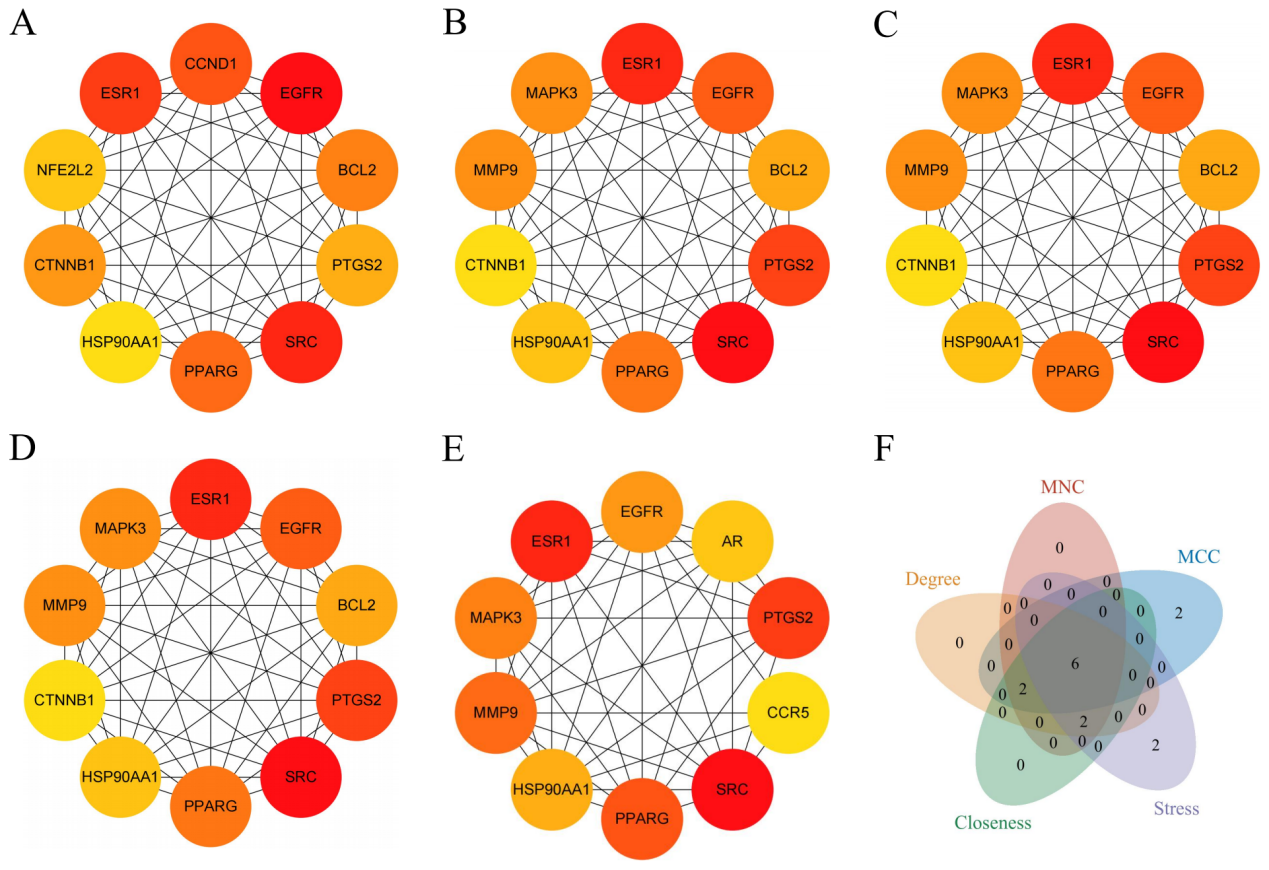


**Supplementary Figure 1.** Screening of core targets. The core target network was based on the MCC (A), MNC (B), Degree(C), Closeness (D), and Stress (E) parameters. (F) Venn diagram of the five algorithms.


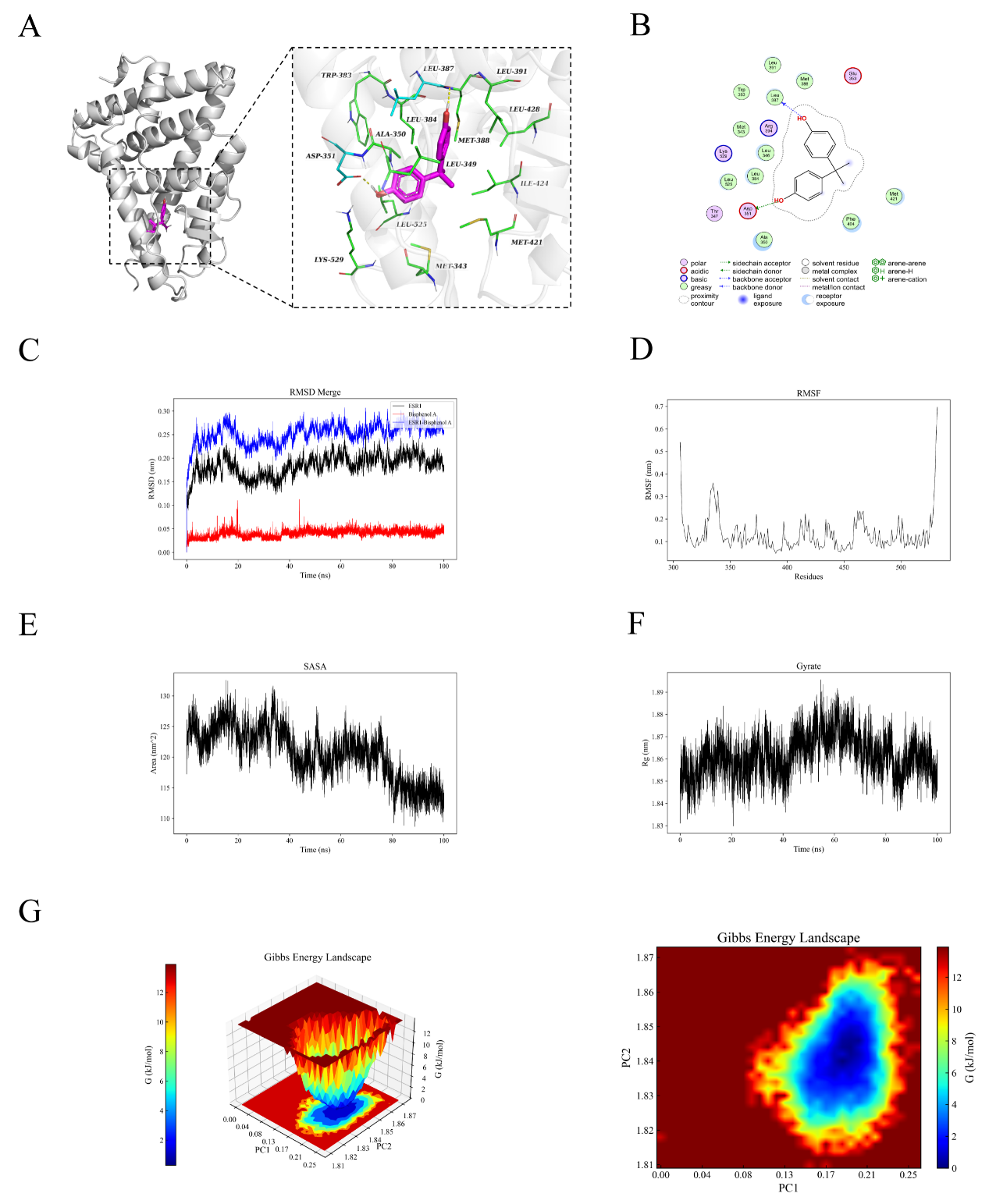


**Supplementary Figure 2.** Molecular docking and dynamics simulation of BPA with ESR1: (A) ESR1-BPA-2D, (B) ESR1-BPA-3D, (C) RMSD values of the ESR1-BPA complex over time, (D) RMSF values of backbone atoms in the ESR1-BPA complex over time, (E) SASA values of the ESR1-BPA complex over time, (F) Rg values of the ESR1-BPA complex over time and (G) FEL of the ESR1-BPA complex.


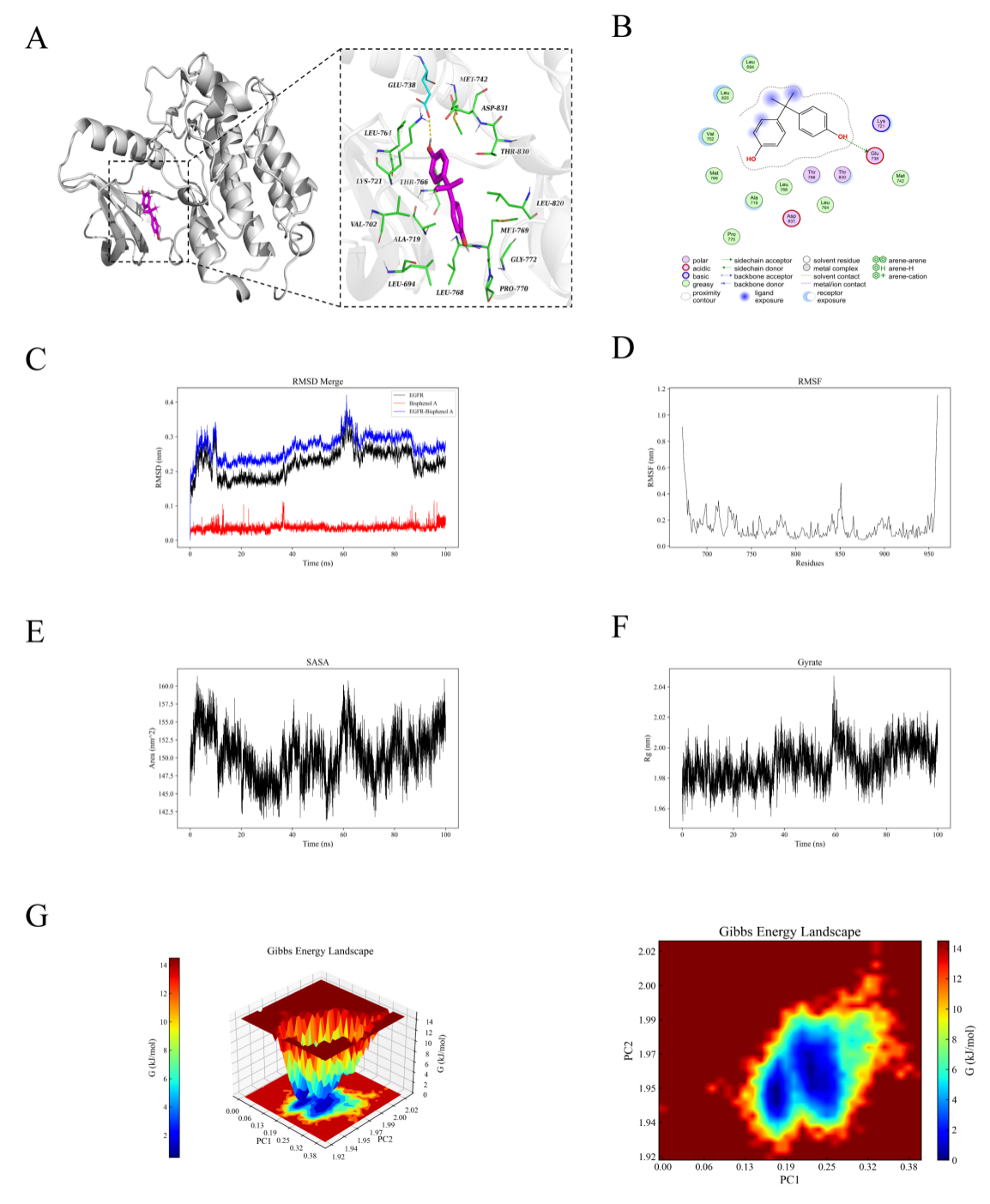


**Supplementary Figure 3.** Molecular docking and dynamics simulation of BPA with EGFR: (A) EGFR-BPA-2D, (B) EGFR-BPA-3D, (C) RMSD values of the EGFR-BPA complex over time, (D) RMSF values of backbone atoms in the EGFR-BPA complex over time, (E) SASA values of the EGFR-BPA complex over time, (F) Rg values of the EGFR-BPA complex over time and (G) FEL of the EGFR-BPA complex.


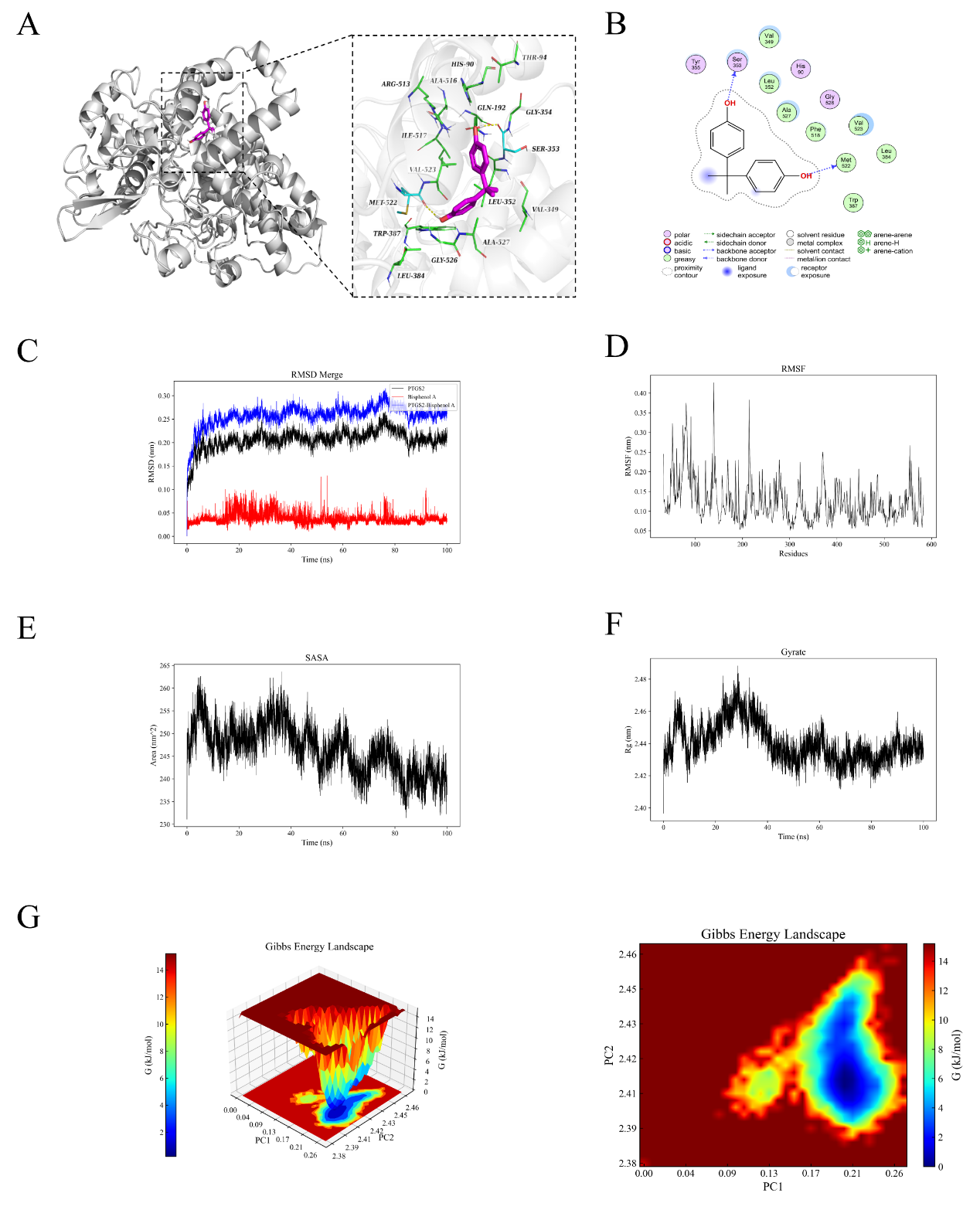


**Supplementary Figure 4.** Molecular docking and dynamics simulation of BPA with PTGS2: (A) PTGS2-BPA-2D, (B) PTGS2-BPA-3D, (C) RMSD values of the PTGS2-BPA complex over time, (D) RMSF values of backbone atoms in the PTGS2-BPA complex over time, (E) SASA values of the PTGS2-BPA complex over time, (F) Rg values of the PTGS2-BPA complex over time and (G) FEL of the PTGS2-BPA complex.


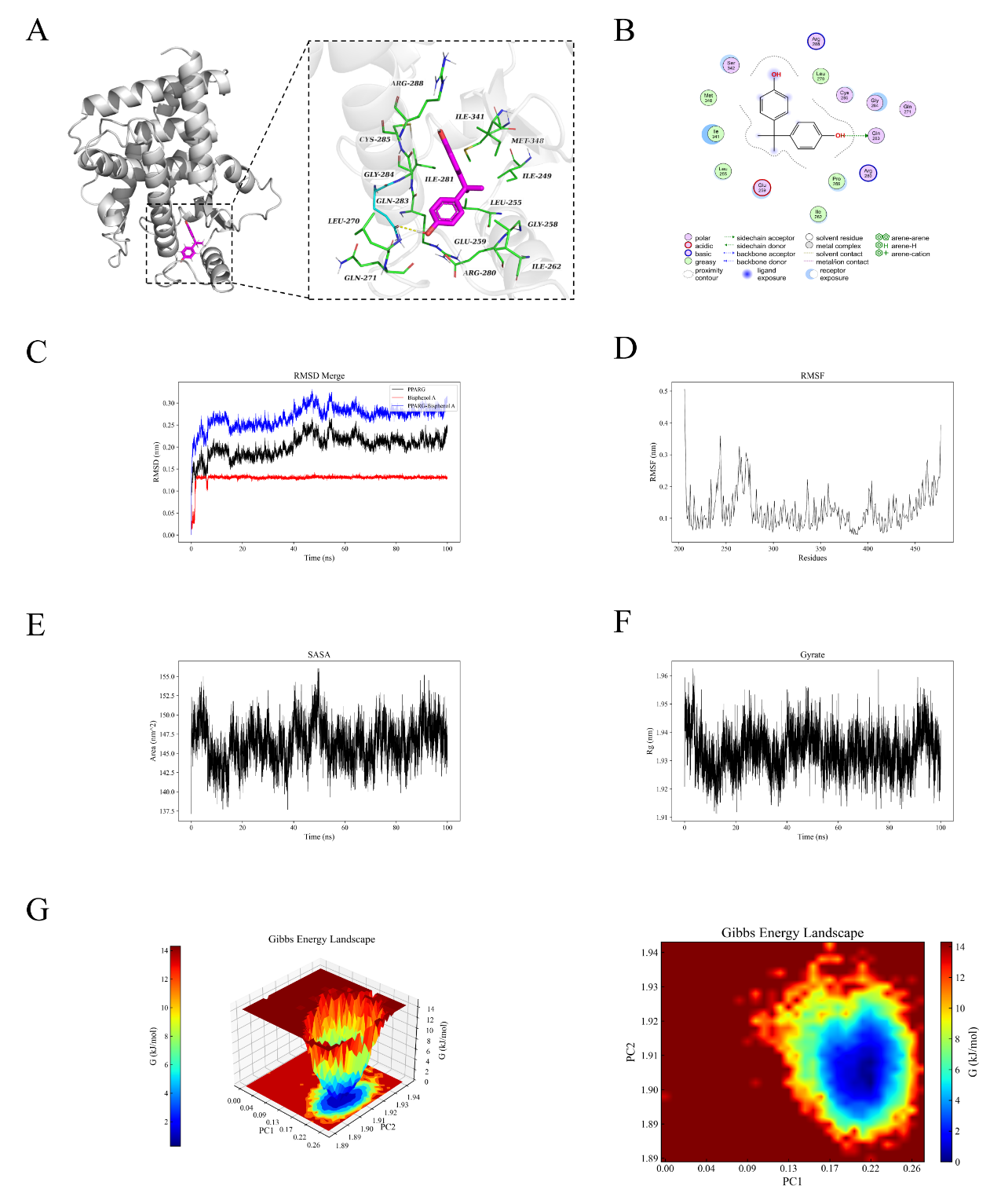


**Supplementary Figure 5.** Molecular docking and dynamics simulation of BPA with PPARG: (A) PPARG-BPA-2D, (B) PPARG-BPA-3D, (C) RMSD values of the PPARG-BPA complex over time, (D) RMSF values of backbone atoms in the PPARG-BPA complex over time, (E) SASA values of the PPARG-BPA complex over time, (F) Rg values of the PPARG-BPA complex over time and (G) FEL of the PPARG-BPA complex.


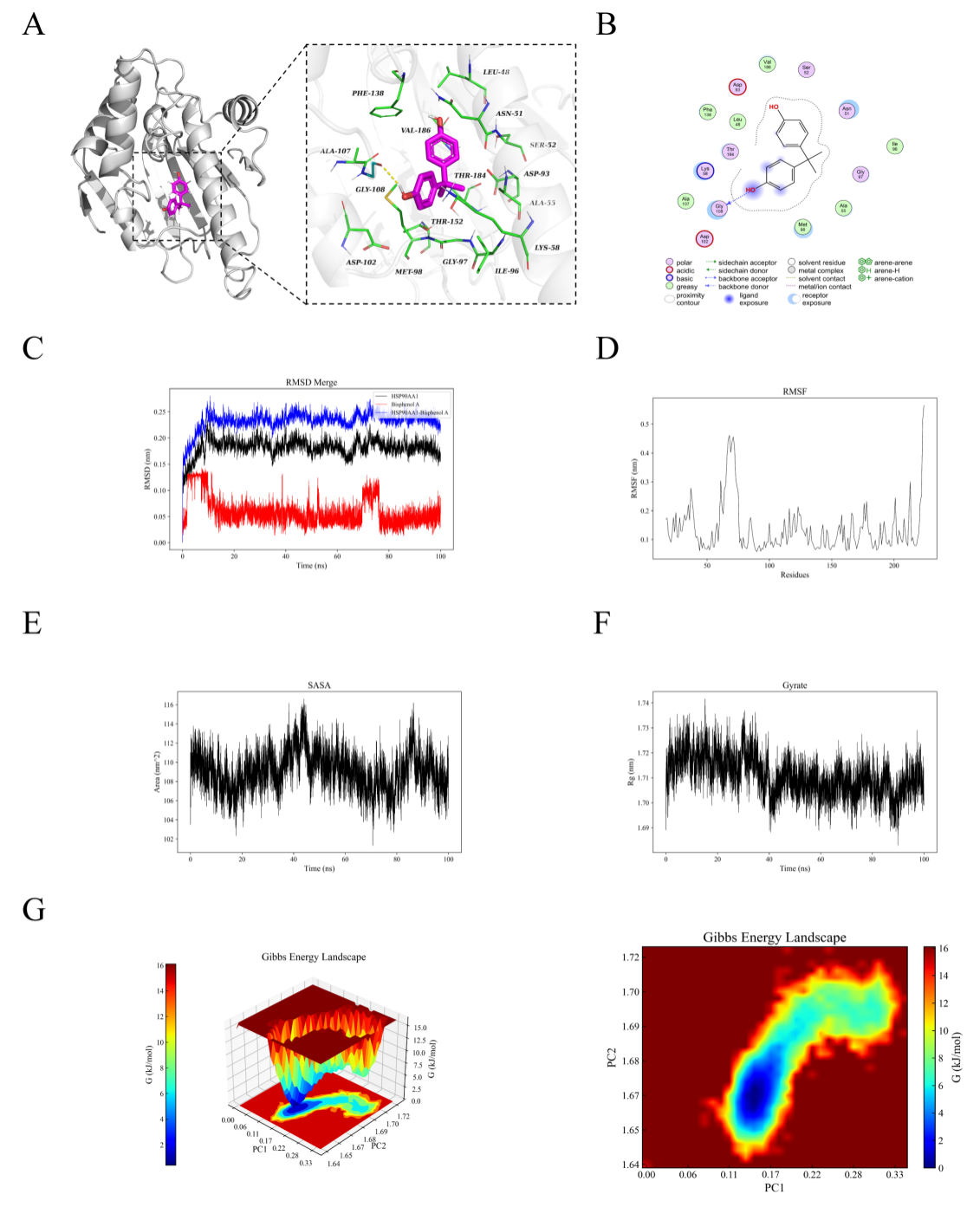


**Supplementary Figure 6.** Molecular docking and dynamics simulation of BPA with HSP90AA1: (A) HSP90AA1-BPA-2D, (B) HSP90AA1-BPA-3D, (C) RMSD values of the HSP90AA1-BPA complex over time, (D) RMSF values of backbone atoms in the HSP90AA1-BPA complex over time, (E) SASA values of the HSP90AA1-BPA complex over time, (F) Rg values of the HSP90AA1-BPA complex over time and (G) FEL of the HSP90AA1-BPA complex.

**Supplementary Table 1.** MM-GBSA parameters for the six protein-ligand complexes.

| **Contribution components** | **SRC** | **ESR1** | **EGFR** | **PTGS2** | **PPARG** | **HSP90AA1** |
| --- | --- | --- | --- | --- | --- | --- |
| Δ_VDWAALS_ | -29.15 ± 0.74 | -29.33 ± 0.32 | -28.14 ± 0.41 | -30.09 ± 0.74 | -28.51 ± 0.03 | -30.92 ± 0.99 |
| ΔE_elec_ | -20.17 ± 0.81 | -29.37 ± 0.04 | -30.95 ± 0.47 | -22.69 ± 0.50 | -21.43 ± 0.02 | -23.75 ± 0.15 |
| ΔE_GB_ | 24.46 ± 1.57 | 31.05 ± 0.02 | 32.11 ± 0.14 | 26.69 ± 0.19 | 20.73 ± 0.15 | 29.24 ± 1.27 |
| ΔE_surf_ | -4.51 ± 0.01 | -4.59 ± 0.02 | -4.73 ± 0.06 | -4.55 ± 0.01 | -4.52 ± 0.05 | -4.27 ± 0.06 |
| ΔG_gas_ | -49.32 ± 1.10 | -58.70 ± 0.32 | -59.09 ± 0.62 | -52.78 ± 0.89 | -49.94 ± 0.03 | -54.67 ± 1.00 |
| ΔG_solvation_ | 19.95 ± 1.57 | 26.46 ± 0.02 | 27.38 ± 0.15 | 22.15 ± 0.19 | 16.21 ± 0.16 | 24.97 ± 1.28 |
| ΔTotal | -29.37 ± 1.01 | -32.24 ± 0.32 | -31.71 ± 0.64 | -30.64 ± 0.91 | -33.73 ± 0.16 | -29.69 ± 1.62 |
